# Late‐life exercise mitigates skeletal muscle epigenetic aging

**DOI:** 10.1111/acel.13527

**Published:** 2021-12-21

**Authors:** Kevin A. Murach, Andrea L. Dimet‐Wiley, Yuan Wen, Camille R. Brightwell, Christine M. Latham, Cory M. Dungan, Christopher S. Fry, Stanley J. Watowich

**Affiliations:** ^1^ Molecular Muscle Mass Regulation Laboratory Department of Health, Human Performance, and Recreation Exercise Science Research Center University of Arkansas Fayetteville Arkansas USA; ^2^ Cell and Molecular Biology Program University of Arkansas Fayetteville Arkansas USA; ^3^ The Center for Muscle Biology University of Kentucky Lexington Kentucky USA; ^4^ Department of Biochemistry and Molecular Biology University of Texas Medical Branch Galveston Texas USA; ^5^ Department of Physiology College of Medicine University of Kentucky Lexington Kentucky USA; ^6^ Department of Athletic Training and Clinical Nutrition College of Health Sciences University of Kentucky Lexington Kentucky USA; ^7^ Department of Physical Therapy College of Health Sciences University of Kentucky Lexington Kentucky USA

**Keywords:** Horvath clock, PoWeR, rDNA, *Rbm10*, *Timm8a1*

## Abstract

There are functional benefits to exercise in muscle, even when performed late in life, but the contributions of epigenetic factors to late‐life exercise adaptation are poorly defined. Using reduced representation bisulfite sequencing (RRBS), ribosomal DNA (rDNA) and mitochondrial‐specific examination of methylation, targeted high‐resolution methylation analysis, and DNAge™ epigenetic aging clock analysis with a translatable model of voluntary murine endurance/resistance exercise training (progressive weighted wheel running, PoWeR), we provide evidence that exercise may mitigate epigenetic aging in skeletal muscle. Late‐life PoWeR from 22–24 months of age modestly but significantly attenuates an age‐associated shift toward promoter hypermethylation. The epigenetic age of muscle from old mice that PoWeR‐trained for eight weeks was approximately eight weeks younger than 24‐month‐old sedentary counterparts, which represents ~8% of the expected murine lifespan. These data provide a molecular basis for exercise as a therapy to attenuate skeletal muscle aging.

## Graphical abstract

Aged mice (22‐24 months old) subjected to a voluntary high‐volume hypertrophic exercise training stimulus (progressive weighted wheel running, or PoWeR) had a lower DNA methylation age in skeletal muscle, as well as a more youthful overall methylation profile.

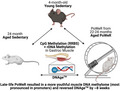

**Abbreviations**
FDR: false discovery rate
mtDNA: mitochondrial DNA
NAD: nicotinamide adenine dinucleotide
PoWeR: progressive weighted wheel running
Rbm10: ribosomal binding motif protein 10
rDNA: ribosomal DNA
RRBS: reduced representation bisulfite sequencing
TCA: tricarboxylic acid cycle
Timm8a1: translocase of inner mitochondrial membrane 8A1

## 1 INTRODUCTION

All tissues, including skeletal muscle, undergo DNA methylation alterations across the lifespan (Turner et al., 2020; Sailani et al., 2019) that may contribute to structural and functional decline with aging. Exercise training alters muscle DNA methylation (Wen et al., 2021), but whether it causes the aged mouse skeletal muscle methylome to more closely resemble that of a younger mouse remains unclear. Using the high‐volume resistance/endurance exercise of progressive weighted wheel running (PoWeR) developed by our laboratory (Murach et al., 2020), mice were trained from 22–24 months of age. After training, we assessed how exercise affected epigenetic aging in skeletal muscle with RRBS, high‐resolution targeted analyses, and a high‐coverage analysis of >500 tissue‐specific murine CpG loci (DNAge™ analysis) (Chew et al., 2018; Kemp et al., 2020; Hayano et al., 2019) that overlaps with the Horvath pan‐tissue epigenetic aging clock (Horvath, 2013). We hypothesized that late‐life combined resistance/endurance exercise would reduce aging‐associated hypermethylation (Turner et al., 2020) and DNAge™ in skeletal muscle.

## 2 RESULTS AND DISCUSSION

**FIGURE 1:**
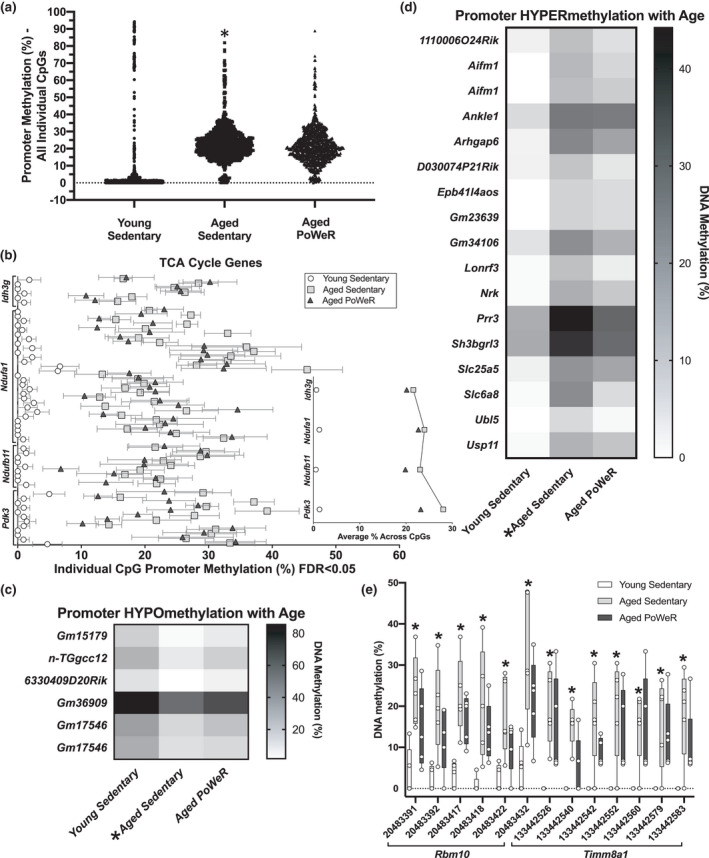
Promoter methylation changes in young, aged sedentary, and aged progressive weighted wheel running (PoWeR) muscles. (a) Percent methylation of promoter CpGs (≤ 1 kb from the transcription start site) in gastrocnemius muscle from aged sedentary versus young mice (all sites *FDR<0.05 aged sedentary versus young; aged PoWeR methylation for the same CpGs shown for reference). (b) Promoters of tricarboxylic acid (TCA) cycle genes hypermethylated with age relative to young mice (all CpG sites *FDR<0.05 aged sedentary versus young, aged PoWeR shown for reference; mean +/‐ SEM); inset shows the average methylation of these CpGs in the promoter. (c) Promoter regions of genes hypomethylated in muscle of aged sedentary mice relative to young mice (*FDR<0.05), but not hypomethylated in aged PoWeR mice relative to young mice. (d) Promoter regions of genes hypermethylated in muscle of aged sedentary mice relative to young mice (*FDR<0.05) but not hypermethylated in aged PoWeR mice relative to young mice. (e) Promoter region methylation of *Rbm10* and *Timm8a1*; x‐axis represents the chromosomal position of individual CpG loci in the promoter region of the gene (*FDR<0.05 aged sedentary relative to young mice). *N* = 5 per group; line at median in (e). Repeated gene names = multiple CpG sites, see supplementary tables for CpG locations. A generalized linear model accounting for all groups was used to determine differential methylation, with a correction for multiple comparisons by controlling false discovery rate (FDR) using the Benjamini–Hochberg method (*α* = 0.05)

RRBS was performed on skeletal muscle and analyzed as described previously (Park et al., 2014; Wen et al., 2021). In the gastrocnemius muscle of sedentary 24‐month‐old mice, 103 unique CpG sites in promoter regions (i.e., within 1 kb upstream of the transcription start site) that mapped to at least one of 9 gene identifiers were hypomethylated compared to 4‐month‐old mice (FDR<0.05, Table S1a), whereas 762 distinct CpG sites that mapped to one or more of 133 different genes were hypermethylated (FDR<0.05, Figure 1a, Table S1b). Pathway analysis of genes with hypermethylated promoters in aged muscle revealed over‐representation in tricarboxylic acid cycle (TCA) regulation (*p *= 0.00572, *q *= 0.125), particularly genes associated with NAD activity (Figure 1b). This may explain the widespread reduction of TCA cycle proteins recently reported in aged human muscle using exploratory proteomics (Ubaida‐Mohien et al., 2019). In exons, 68 CpG sites that mapped to at least one of 27 genes were hypomethylated (Table S2a), while 864 distinct CpG sites that mapped to one or more of 146 genes were hypermethylated in aged sedentary muscle relative to young (Table S2b, FDR<0.05). The same pattern occurred with intron methylation in aged sedentary relative to young muscle; 271 CpG sites that mapped to at least one of 131 genes were hypomethylated (Table S3a); and 2,261 CpG sites that mapped to one or more of 301 genes were hypermethylated (Table S3b, FDR<0.05). No genes were hypomethylated in all three regions with age, while 18 genes had hypermethylation in all three regions (gene list in Table S3c).

In promoters, relative to young sedentary mice, five genes that contained at least one hypomethylated CpG in aged sedentary muscle did not contain any significantly hypomethylated CpGs in mice that engaged in PoWeR from 22–24 months of age (genes mapped to six CpGs, Figure 1c, Table S4a). Eighteen genes that had at least one hypermethylated CpG in their promoter with age alone did not have any significantly hypermethylated CpGs in aged PoWeR muscle relative to young (29 CpGs mapped to these genes, Figure 1d,e). In multiple CpGs, PoWeR attenuated age‐associated promoter region hypermethylation of *Rbm10* (Figure 1e), a pleiotropic factor implicated in: (1) alternative splicing (Loiselle & Sutherland, 2018) which is generally dysregulated by aging in skeletal muscle (Ubaida‐Mohien et al., 2019), (2) regulation of survival of motor neuron (SMN) alternative splicing (Sutherland et al., 2017), a protein that can control muscle weight and function throughout the lifespan (Zhao et al., 2021), and (3) striated muscle hypertrophy (Mohan et al., 2018). PoWeR was also associated with relatively lower methylation across the promoter of *Timm8a1* in aged mice (Figure 1e). In skeletal muscle, a PGC‐1β knockout model that exhibits impaired mitochondrial function and oxidant defense is associated with reduced *Timm8a1* levels (Ramamoorthy et al., 2015), while loss of *Timm8a1* function results in swollen mitochondria and broken cristae (Song et al., 2021). Using high‐resolution targeted methylation analysis (>1,000x coverage per CpG on average), we confirmed that promoter regions of *Rbm10* and *Timm8a1* were less hypermethylated with exercise in aged muscle (Figure S1a,b). In addition, promoters of 9 genes had a unique hypomethylated CpG (none had multiple) and 10 genes had one or more unique hypermethylated CpGs in aged PoWeR relative to young muscle (9 and 12 CpGs mapped to these genes, respectively, FDR<0.05); these promotors were not affected by aging alone (Table S4b). Proportionally, more CpGs hypomethylated with age were affected by PoWeR than those hypermethylated by age, but a larger absolute number of CpGs hypermethylated with age were affected by PoWeR since aging was more associated with hypermethylation. Exon data are reported in Figure S2 and Table S5, and intron data are found in Table S6. Introns followed a different pattern compared with the rest of the genome with respect to exercise mitigating the epigenetic effects of aging. Relative to young mice, a comparatively large number of genes (93) had intronic regions with at least one CpG hypomethylated by agingbut no CpGs hypomethylated in aged PoWeR muscle (genes mapped to 112 CpGs).

Transcription can be controlled by methylation at a single CpG or by clusters of CpGs, called “CpG islands”. Analysis of CpG islands (FDR<0.05) generally reflected the individual site data. Sixteen genes (which mapped to 12 CpG islands) were hypermethylated in aged animals but not in aged PoWeR relative to young, while five genes (which mapped to four CpG islands) were hypermethylated only in aged PoWeR mice. Eight genes (which mapped to six CpG islands) were hypomethylated by age but not by PoWeR in aged mice relative to young; among these was *Hoxa3*. *Hox* genes were recently implicated as hotspots for age‐associated methylation changes in muscle (Turner et al., 2020; Voisin et al., 2021). Three genes (which mapped to two CpG islands) were hypomethylated only in aged PoWeR mice. The CpG island analyses are presented in Table S7.

**FIGURE 2:**
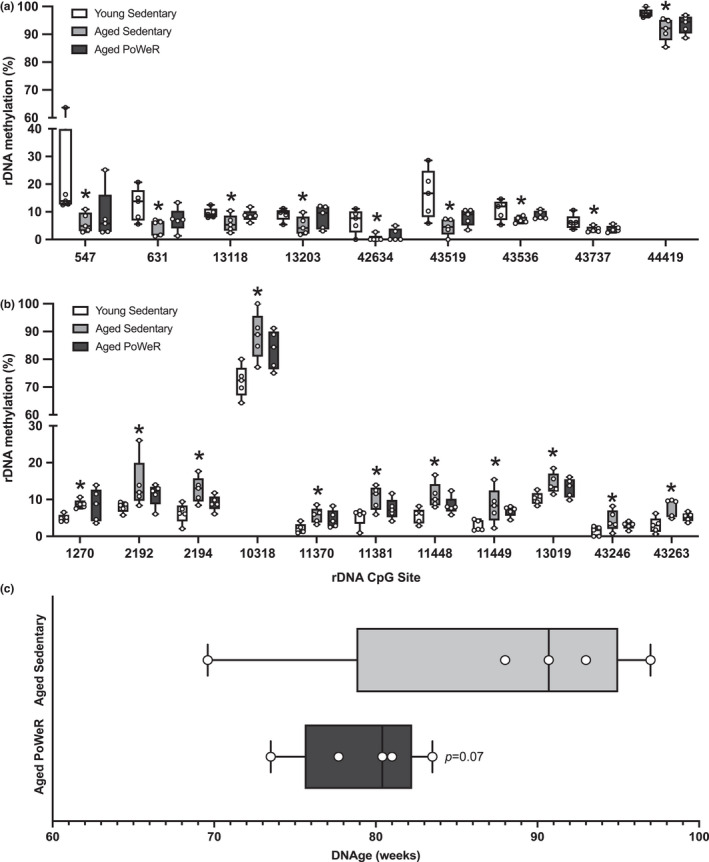
Ribosomal DNA (rDNA) methylation and DNAge™ analysis. (a) rDNA CpGs (listed by chromosomal position) hypomethylated in muscle from aged sedentary versus young animals (*FDR<0.05), but not hypomethylated in muscle from aged PoWeR versus young animals. (b) rDNA CpGs (listed by chromosomal position) hypermethylated in muscle from aged sedentary versus young animals (*FDR<0.05), but not hypermethylated in muscle from aged PoWeR versus young animals. (c) DNAge™ analysis of muscle from aged sedentary versus aged PoWeR muscle, analyzed using a directional *t*‐test. A generalized linear model accounting for all groups was used to determine differential methylation in (a) and (b), with a correction for multiple comparisons by controlling false discovery rate (FDR) using the Benjamini–Hochberg method (*α* = 0.05); histograms depict median with a line

rDNA is hypermethylated with age and harbors a highly conserved methylation clock of aging (Wang & Lemos, 2019). We found 360 CpG sites in rDNA that were differentially methylated in aged relative to young muscle (FDR<0.05). Of these sites, 15 were hypomethylated and 345 were hypermethylated (Table S8a,b). Nine sites hypomethylated in sedentary aged relative to young muscle were not hypomethylated after PoWeR (Figure 2a). Eleven sites hypermethylated in aged relative to young muscle were shifted toward youthful methylation levels by PoWeR (Figure 2b). Using targeted high‐resolution methylation analysis (>10,000x coverage per rDNA CpG on average), methylation at and around an enhancer region site (CpG 43519) was demonstrated to be higher with exercise relative to aging alone, but these sites were hypermethylated relative to young irrespective of exercise (Figure S1c). Our general conclusion from RRBS that exercise altered rDNA methylation in aged muscle is valid, but the targeted analysis highlighted the potential influence of read coverage on absolute methylation levels (RRBS coverage at site 43519 was 23x on average). The majority of unique differentially methylated rDNA sites in aged PoWeR relative to young sedentary muscle were hypermethylated (78 out of 85), pointing to a distinct interaction between aging and exercise with respect to rDNA regulation (Table S9). Muscle rDNA methylation alterations with aging and exercise may have implications for ribosome biogenesis, a process induced during muscle hypertrophy Figueiredo et al. (2021). In our dataset, mitochondrial DNA (mtDNA) methylation coverage was generally low, and of the sites with ≥10x coverage in each animal (33 CpGs), none were altered by age or age and PoWeR (data not shown).

Several mammalian epigenetic aging clocks have been developed with the aim of expediting the discovery and validation of therapeutics and interventions to attenuate, prevent, or reverse biological aging (Simpson & Chandra, 2021). Zymo Research's validated DNAge™ algorithm, which expands upon the Horvath pan‐tissue clock built using elastic net regression (Horvath, 2013) and is accurate in murine muscle, was used to compare the chronological age of muscle from aged sedentary and PoWeR animals to that of young animals. Despite one sedentary mouse that had an aberrantly young predicted age (with no reason to exclude it based on the behavior/appearance of the mouse or tissue, or anything anomalous according to the principle coordinate analysis plot), the epigenetic age of PoWeR muscle was 10% lower (~8 weeks younger) compared to sedentary (PoWeR = 79.2 wks [SD, 3.8 wks], aged sedentary = 87.7 wks [SD, 10.6 wks], young sedentary = 10.4 wks [SD, 9.8 wks]) (Figure 2c, Table S10). Aging generally results in greater molecular variability or “disorderliness”, and it is notable that PoWeR resulted in lower variability in the DNAge™ estimate of older animals (Figure 2c). If the aged sedentary mouse with the lowest methylation age were removed, the epigenetic age difference between aged exercised and aged sedentary muscle increases to ~13 weeks (*p *= 0.007). Ribosomal DNAge clock analysis (i.e., rDNAge) (Wang & Lemos, 2019) showed a 9% reduction with PoWeR, but this was not statistically significant (*p *= 0.29, *t* = 0.5786; data not shown). Shannon entropy (Hannum et al., 2013) of nuclear and rDNA methylation was higher with aging (FDR<0.05) and not influenced by PoWeR, but mtDNA was similar between young sedentary and aged PoWeR (Figure S3).

DNAge™ was sufficiently sensitive to detect a younger epigenetic age in mouse gastrocnemius muscle after 8 weeks of PoWeR, but recent advancements promise improved robustness and accuracy of muscle‐specific methylation‐based aging clocks (Voisin et al., 2020). Future studies may clarify which exercise‐mediated effects on DNAge occur independent of aging. In some instances, chronological age is less associated with muscle dysfunction than other related factors such as body mass or cardiorespiratory fitness (Distefano et al., 2017). Nevertheless, the attenuation of muscle epigenetic aging by exercise supports recent targeted observations in humans (Blocquiaux et al., 2021; Ruple et al., 2021) and adds to the growing body of evidence touting exercise as a strategy to extend healthspan. Our work provides potentially modifiable epigenetic markers for improving muscle health with age once the mechanistic bases of dynamic DNA methylation alterations in muscle fibers are more clearly defined (Small et al., 2021).

## CONFLICT OF INTEREST

SJW is the Founder of Ridgeline Therapeutics and, since manuscript submission, ALD‐W has become an employee of Ridgeline Therapeutics. YW is sole proprietor of Myoanalytics LLC. No other conflicts are declared.

## AUTHOR CONTRIBUTIONS

Research was conceived by KAM, CSF, and SJW. Experiments were carried out by CRB, CML, and CMD. Data were analyzed by KAM, ALD‐W, and YW. Manuscript was written and figures were generated by KAM, ALD‐W, and YW. Funding support was provided by KAM, CSF, and SJW. All authors reviewed, edited, and approved of the final manuscript.

## Supporting information

Fig S1

Fig S2

Fig S3

Table S1

Table S2

Table S3

Table S4

Table S5

Table S6

Table S7

Table S8

Table S9

Table S10

Table S11

Table S12

Table S13

Supplementary Material

## Data Availability

All sequencing data are made available through GEO: GSE175410, and processed raw data are presented in supplemental tables.
