# Supplementary figures and images for: Late‐life exercise mitigates skeletal muscle epigenetic aging
Source: Aging Cell. 2021 Dec 21;21(1):e13527. doi: 10.1111/acel.13527 (PMC8761012; doi:10.1111/acel.13527)

**Figure S1**

**A**

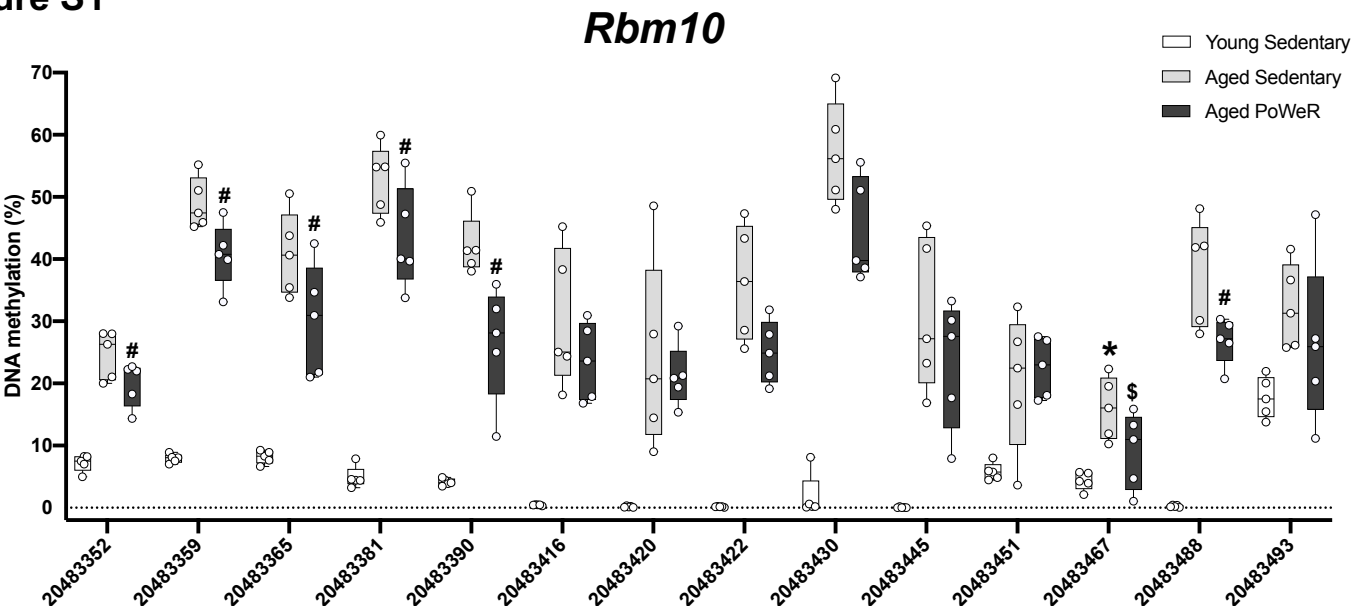

**B**

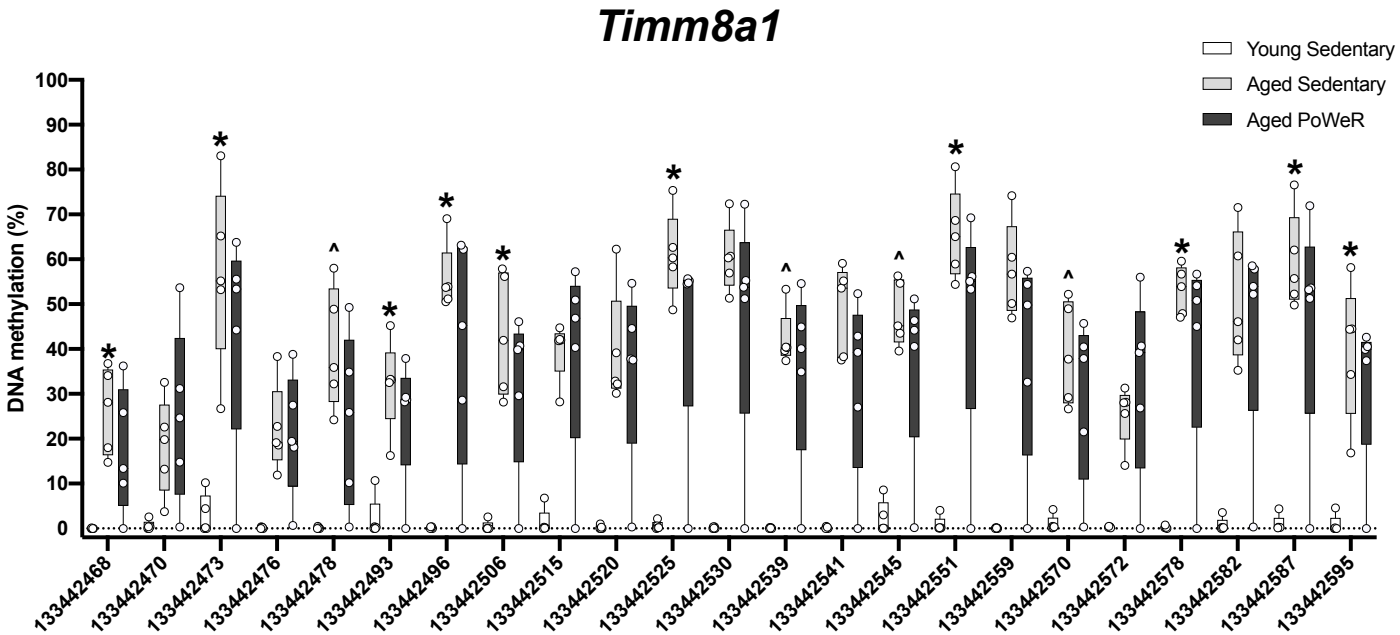

**C**

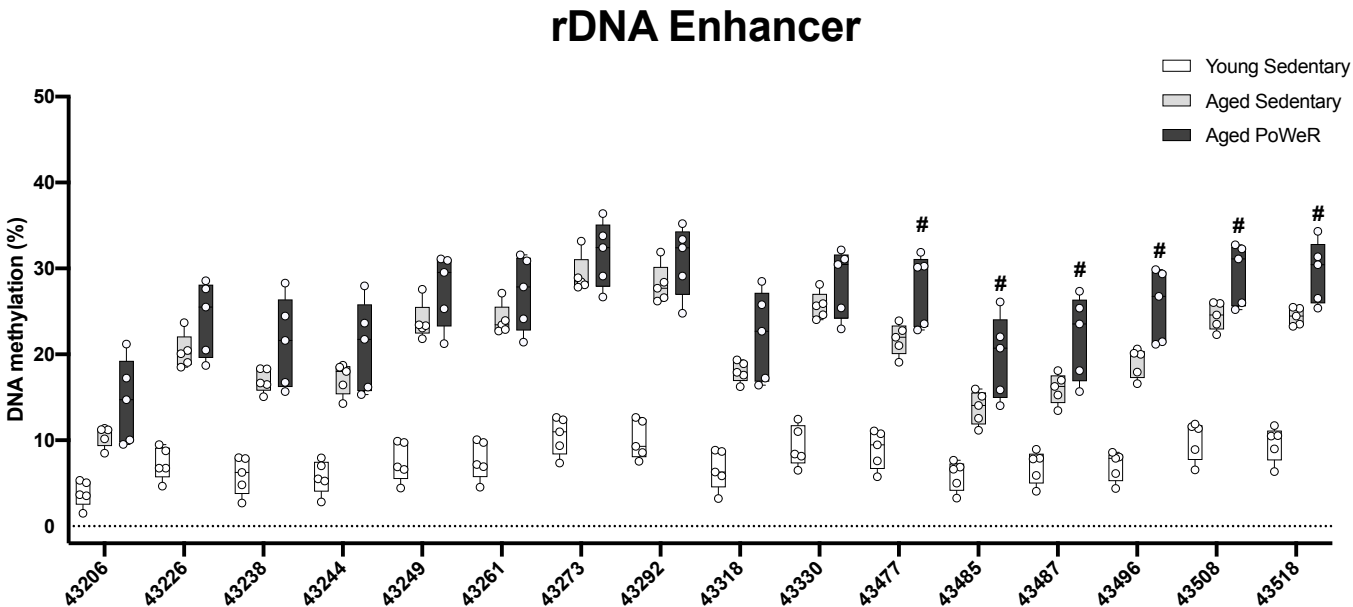

Supplement: Supplementary file 1 — Fig S1 [file ACEL-21-e13527-s012.pdf]

Figure S2

A

EXON HYPOmethylation with Age

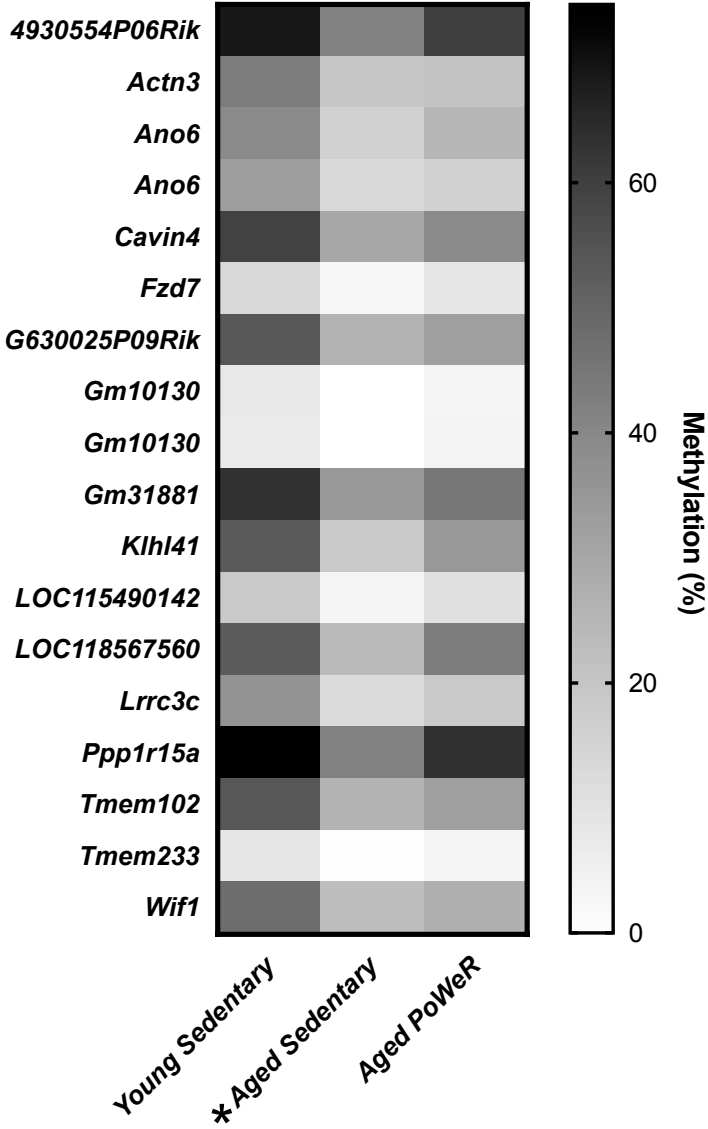

B

EXON HYPERmethylation with Age

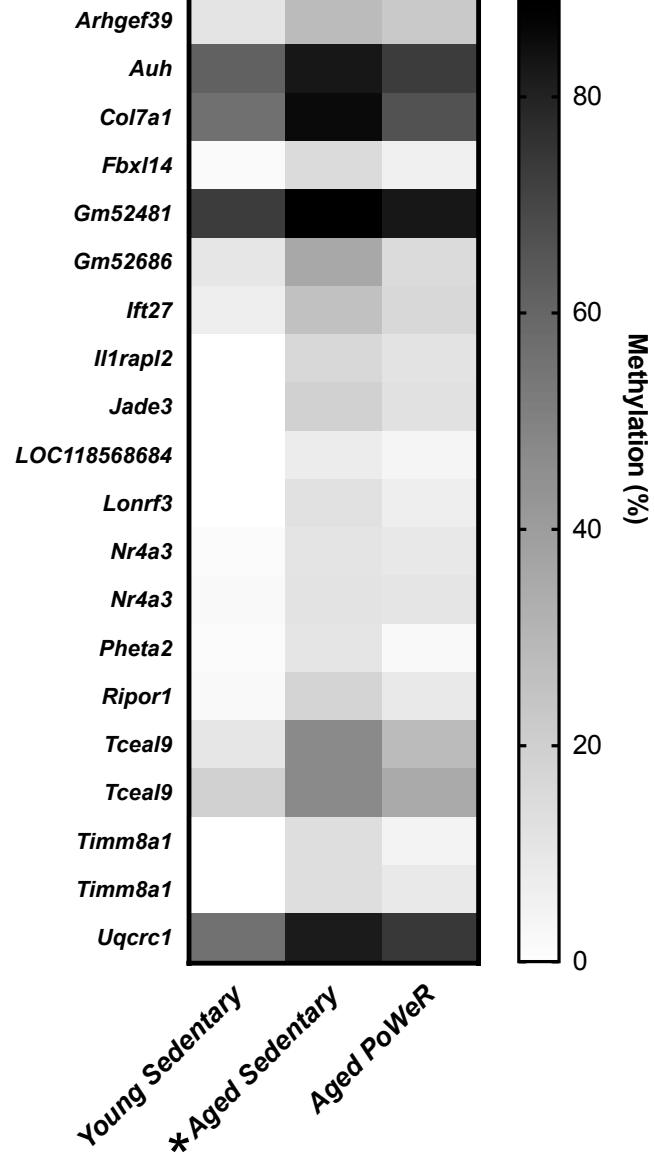

Supplement: Supplementary file 2 — Fig S2 [file ACEL-21-e13527-s011.pdf]

Figure S3

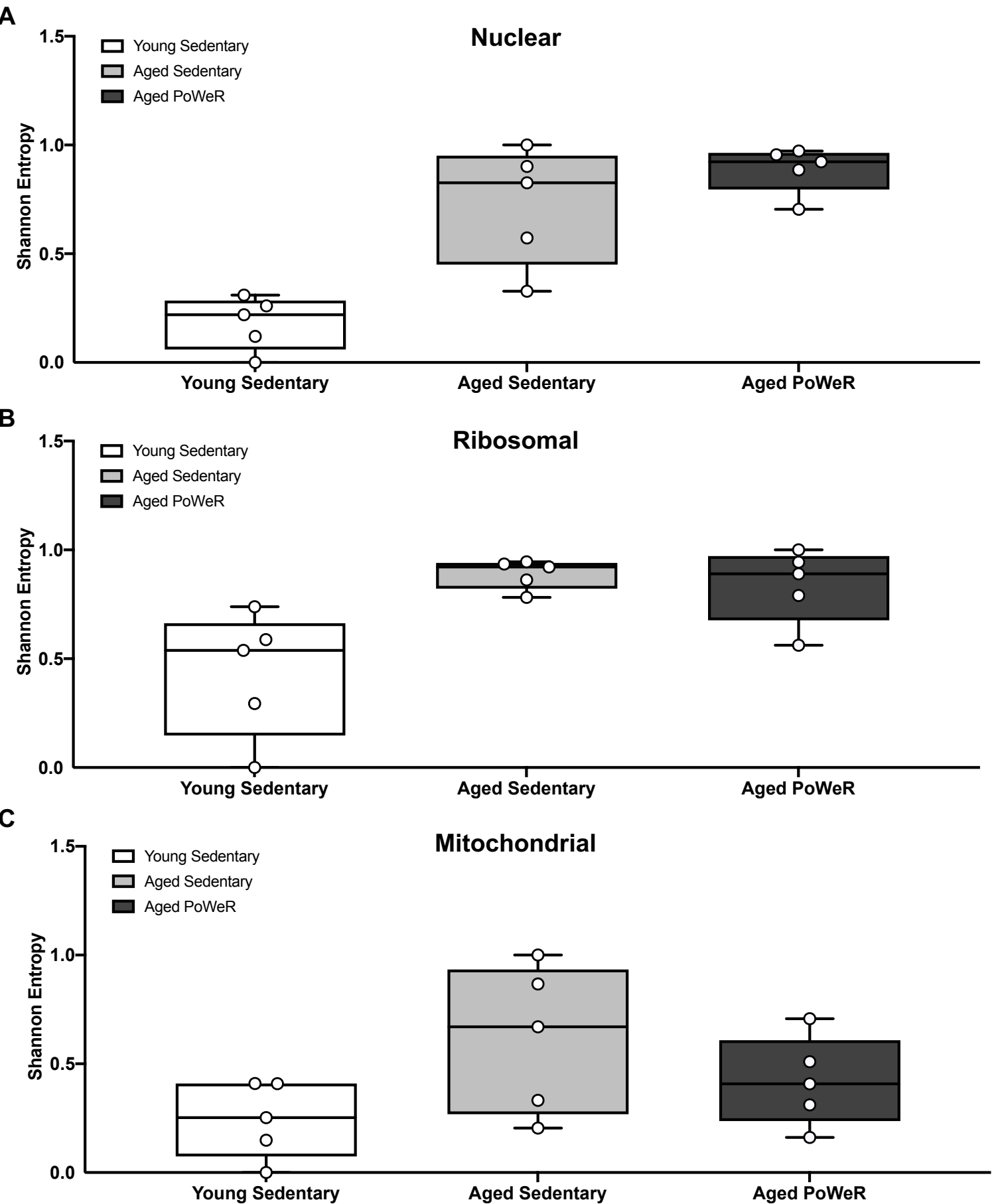

Supplement: Supplementary file 3 — Fig S3 [file ACEL-21-e13527-s015.pdf]
