# Supplementary material for: Late‐life exercise mitigates skeletal muscle epigenetic aging
Source: Aging Cell. 2021 Dec 21;21(1):e13527. doi: 10.1111/acel.13527 (PMC8761012; doi:10.1111/acel.13527)
Supplement: Supplementary file 17 — Supplementary Material [file ACEL-21-e13527-s001.docx]

**Appendix S1 Methods**

**Ethical approval**

Animal procedures were approved by the Institutional Animal Care and Use Committee at the University of Kentucky (protocol #2019-3301). All mice were singly housed in the same temperature- and humidity-controlled room on a 12h:12h light-dark cycle. Mice were given *ad libitum* access to food and water and were euthanized by cervical dislocation under deep anesthesia.

**Experimental design**

Approximately 22-month-old female C57BL/6 mice were obtained from the Charles River Laboratories National Institute of Aging research colony; exact ages are not recorded for this colony, but birth month is documented. Sedentary, 4-month-old female mice were used as a young comparator. Aged mice were assigned to the PoWeR or sedentary control group (n=5 per group). Mice in the PoWeR group were singly housed in cages (37x15x13 mm LxWxH) with running wheels to allow for monitoring of individual running distance using ClockLab software (Actimetrics, v6.1.01), and mice in the sedentary group were singly housed in cages without running wheels. Following an introductory week with an unweighted wheel, 8 weeks of PoWeR training commenced with the following weight progression: 2g in week 1, 3g in week 2, 4g in week 3, and 5g in weeks 4-8. One-gram magnets (product no. B661, K&J Magnetics) were affixed to one side of the wheel to allow for the progressive increase in weight, as previously described (Murach et al. 2020; Wen et al. 2021). The asymmetrical loading pattern of the weight produces an unbalanced wheel, resulting in frequent stopping and restarting of running which forces the mice to overcome the weight of the wheel repeatedly instead of relying on momentum after the initial starting effort. Mice were approximately 24 months old upon completion of the experiment. Since these experiments were run on older mice than previously used for PoWeR, and to facilitate higher volumes of running in these aged mice, the PoWeR protocol was modified to only achieve five grams on the wheel as opposed to six by the final stage of training. Following 8 weeks of PoWeR, or 8 weeks as a sedentary control, mice were humanely euthanized by cervical dislocation under deep anesthesia after a 24-hour wheel lock and overnight fast, and the gastrocnemius muscle was rapidly dissected and flash frozen. PoWeR-trained mice ran an average weekly distance of 6.4 ± 2.6 kilometers (km) per day over 8 weeks (from 22 to 24 months), and neither aged PoWeR (n=5) nor aged sedentary mice (24 months, n=5) underwent changes in bodyweight throughout the study (PoWeR: 26.2 ± 1.4g at 22 months of age to 26.5 ± 1.6g at 24 months of age; sedentary: 28.7 ± 3.9g at 22 months of age to 28.6 ± 3.7g at 24 months of age, *p*=0.148 for group, *p*=0.424 for time; data analyzed with a two-way repeated measures ANOVA (with factors group [PoWeR, sedentary; F=2.566], and time [22 and 24 months of age; F=0.709]). Body weight-adjusted gastrocnemius weight was also not different between aged PoWeR and aged sedentary mice (3.64 mg/g BW [SD, 0.39 mg/g BW] versus 3.24 mg/g BW [SD, 0.49 mg/g BW], respectively, p=0.19, 2-tailed unpaired t-test, t=1.44); a lack of whole-muscle hypertrophy with PoWeR is consistent with our previous work in young mice and is likely explained by exercise training eliciting a transition from fast to relatively slower-twitch fibers (Murach et al. 2020). Bodyweight for the young mice (four months old, n=5) was 24.5g [SD, 1.6g].

**DNA isolation**

DNA was isolated from the gastrocnemius muscle using the Quick-DNA™ Miniprep Plus kit (Zymo Research, D4068) as specified in the protocol. DNA quantity was assessed using NanoDrop^TM^ (Thermo Scientific), and quality was assessed by NanoDrop^TM^ (Thermo Scientific) and TapeStation 4200 (Agilent). RRBS and DNAge™ samples were required to have a 260/280 ratio ≥1.8, and ideally a DNA Integrity Number ≥6.0.

**RRBS analysis of mouse muscle DNA**

RRBS was conducted by Zymo Research on the DNA isolated from the gastrocnemius. Quality and concentration were assessed using a Fragment Analyzer™ (Advanced Analytical Technologies, Inc.). “Classic” RRBS library preparation was performed by digesting 100 ng DNA with 30 units of MspI enzyme (New England BioLabs, R0106), and fragments were ligated to pre-annealed adapters containing 5’-methyl-cyotosine. Adapter-ligated fragments ≥50 base pairs were recovered using the DNA Clean & Concentrator-5™ kit (Zymo Research, D4003) and bisulfite-converted using the EZ DNA Methylation-Lightning Kit™ (Zymo Research, D5030) following the standard protocol. Preparative-scale PCR was performed, and the products were purified again using the Clean and Concentrator kit. Paired-end sequencing was executed with an Illumina HiSeq using the Rapid Run Mode with a paired-end 100 base pair configuration, and sequenced reads from bisulfite-treated libraries were identified using standard Illumina base calling software.

Raw FASTQ files were trimmed for adapters, filled-in nucleotides, and poor quality using TrimGalore 0.6.4 and options for “--rrbs" and “--non_directional” mode, retaining reads with minimum quality above a phred score of 30. FastQC 0.11.8 was used to assess the effect of trimming and overall quality of the data. A custom genome assembly to interrogate both nuclear and rDNA methylation was generated by adding the consensus rDNA repeat sequences, BK000964.3, as a separate chromosome to the mouse (GRCm39) reference genome assembly, as recently described by our laboratory (Wen et al. 2021). The GRCm39 reference assembly included the mitochondrial genome, which was used to determine mtDNA CpG sites. Due to mapping interference from highly similar sequences within the reference assembly, the rDNA sequences were found using the Basic Local Alignment Search Tool (BLAST, NCBI) by comparing the respective rDNA sequences to the reference assembly followed by masking of these sequences in the genome using N’s. Bismark 0.19.0 was used to align to the custom mouse reference genome. Methylated and unmethylated read totals for each CpG site were collected using the Methylation Extractor tool. Methylation levels of each sampled cytosine were estimated as the number of reads reporting a “C”, divided by the total number of reads reporting a “C” or “T”. Differential methylation analyses were performed using the R Bioconductor package, methylSig v1.0.0 (Park et al. 2014), which accounts for both read coverage (minimum set to 10x for each animal) and biological variation. The data were analyzed using a beta-binomial distribution generalized linear model, and for individual CpG data, sites where a CpG was present in every sample were included for analysis. The false discovery rate (FDR, reported as the Benjamini-Hochberg adjusted *p* value) was controlled at α<0.05. Promoters were defined as within 1 kb upstream of transcription start sites (Wen et al. 2021). Multiple unique CpGs were allowed to map to each individual gene identifier, and multiple gene identifiers were allowed to map to one unique CpG since one gene may have many CpGs and a CpG labeled as the promoter of one gene may be part of another gene’s exon, for instance. To determine the effects of PoWeR on aging, all CpG sites in aged sedentary versus young and aged PoWeR versus young with an FDR<0.05 were identified along with their genomic location (promoter, exon, or intron). The gene lists were then split into lists of hypomethylated genes and hypermethylated genes. Each hypomethylated/hypermethylated gene list for the aged sedentary versus young was then compared to that of the aged PoWeR versus young to identify genes that were uniquely hypomethylated or hypermethylated by aging or PoWeR, irrespective of the number of differentially methylated CpG sites. These unique genes were mapped back to individual CpGs used to generate the heat maps, or to CpG islands; multiple instances of CpGs and CpG islands were accounted for such that the CpG/CpG islands numbers represent unique CpGs/CpG islands. Additionally, genes that had simultaneous significantly hypo- and hyper- methylated CpGs were not counted. A parallel analysis was conducted using only CpG sites (not accounting for unique genes), see Tables S11-13. Pathway analysis was conducted using ConsensusPathDB (KEGG, Reactome, Wikipathways) using default settings; genes with hypermethylated CpGs (FDR<0.05) in their promoters in the comparison between the young sedentary and aged sedentary groups (127/133 of which mapped into ConsensusPathDB, and 62 of which were present in at least one pathway) were cross-referenced against the list of genes with at least one CpG in their promoter for at least one animal in these two treatment groups (1,551 of which were present in at least one pathway) for over-representation analysis.

Ribosomal DNA methylation clock (rDNAge) was first described by Wang and Lemos in 2019, which included 72 CpG sites in the rDNA sequence. These CpG sites and their corresponding weights along with the intercept were obtained from Supplemental Table S2 of their manuscript (Wang and Lemos, 2019). All 72 CpG sites had greater than 10x coverage in our dataset. The rDNAm age was calculated for each mouse using the following equation:

$$\mathrm{rDNAm}=e^{(\varepsilon+\sum_{i=1}^{n} \omega_{i}x_{i})}$$

where $x_{i}$ is the methylation level of the $i$th CpG site, and $\omega_{i}$ is the weight for the $i$th CpG site, $n=72$ CpG sites of the model, and $\varepsilon$ is the intercept. The concept of Shannon entropy was introduced in 1963 by Shannon and Weaver and adapted to methylation data based on the Bernoulli distribution, which assumed maximum entropy at the 50% methylation level. We employed a modified version of the previously described genome-wide methylation entropy analysis (Hannum et al., 2013). We computed the methylation entropy for the CpG sites in the nuclear genome, mitochondrial genome, and the rDNA separately using the following equation:

$entropy=\frac{-1}{{log}_{2}N}\sum_{i=1}^{N} \left( x_{i}{log}_{2}x_{i}-(1-x_{i}){log}_{2}(1-x_{i}) \right)$

where $x_{i}$ is the fraction of methylated reads for the $i$th CpG site, and $N$ is the total number of CpG sites within the nuclear, mitochondrial, or ribosomal DNA sequences. The entropy is subsequently normalized to between 0 and 1 based on the minimum and maximum entropy values.

**Targeted bisulfite sequencing of mouse muscle DNA**

Isolated DNA was processed and analyzed using Zymo Research’s MethylCheck™ service, which designs primers with Rosefinch (a proprietary tool for sodium bisulfite converted DNA-specific primer design). Primer design set amplicons between 100-300bp and annealing outside the region of interest. Primers were resuspended or ordered in TE solution at 100μM, then diluted to 2μM and tested with real-time PCR (RT-PCR) using 1 ng of bisulfite-converted control DNA in duplicated individual reactions. A DNA melt analysis confirmed that there was a specific PCR product for each primer by requiring: 1) duplicated reactions did not have a difference in crossing point value that exceeded one, 2) that melting curves fell in the expected range for the PCR product of interest, and 3) that duplicated melts displayed a coefficient of variation of less than 10% in their primer melting temperatures (TMs). Validated primers used bisulfite-converted DNA samples, generated anew with the EZ DNA Methylation-Lightning™ Kit (Zymo Research, D5030) according to the manufacturer’s instructions were used to perform multiplex amplification via the Fluidigm Access Array™ System following the manufacturer’s instructions, and amplicons were pooled and barcoded following Fluidigm’s protocols. Barcoded samples were then purified using Zymo Research’s DNA Clean & Concentrator-5™ kit, prepped for massively parallel sequencing with a MiSeq V2 300bp Reagent Kit (Illumina), and analyzed using a paired-end sequencing protocol that followed manufacturer guidelines on an Illumina MiSeq. As with RRBS, standard Illumina base-calling software was used. Subsequently, a proprietary Zymo Research Python pipeline was used to trim low quality nucleotides and adapter sequences. Sequence reads were aligned to the GRCm39 reference genome assembly using Bismark 0.14.3, and the paired-end alignment setting required read 1 and read 2 to be aligned within a 0-1000bp window. Index files were generated using the “bismark_genome_preparation” command and the entire GRCm39 genome assembly; the “--non_directional” parameter was also applied but all other Bismark parameters were set to default. Nucleotides found in primers were trimmed from the amplicons during methylation calling. As with the DNAge analysis^™^, methylation levels for each cytosine were calculated by dividing the number of reads reporting a “C” by the number of reads reporting a “C” or “T.”

**DNAge^™^ Analysis**

DNAge™ was performed by Zymo Research on the DNA isolated from the gastrocnemius muscle with the Quick-DNA™ Miniprep Plus kit (Zymo Research, Cat. No. D4068). The EZ DNA Methylation-Lightning™ Kit (Zymo Research, Cat. No. D5030) following the standard protocol was used for bisulfite conversion. After enriching samples specifically for the sequencing of >500 age-associated gene loci via the Simplified Whole-panel Amplification Reaction Method (SWARM™) (Kemp et al. 2020), locus-specific (single-index) primers were used to target and amplify the loci of interest (loci known to be relevant to epigenetic aging, listed in Table S10). Select-a-size (Zymo Research, Cat. No. D4080) was used for cleanup after PCR, and then AMPure beads were used for size selection. Sequencing was run on an Illumina HiSeq instrument with a paired-end 100bp read length configuration, and sequences were identified by Illumina base calling software then aligned to the mm10 genome using Bismark. Reads with a phred quality score above 20 were removed, and TrimGalore was used to remove adapters and low-quality data. Methylation levels for each cytosine were calculated by dividing the number of reads reporting a “C” by the number of reads reporting a “C” or “T.” The percentage of methylation for these specific sequences of interest were then put into Zymo’s proprietary DNAge^™^ predictor that is validated in muscle (Hayano et al. 2019), which had been established using elastic net regression in collaboration with Dr. Steve Horvath (Chew et al. 2018), and has recently been employed and independently validated across various experimental designs (Kemp et al. 2020; Hayano et al. 2019), to determine the DNAge^™^. A penalized regression model’s coefficients *b*_0_, *b*_1_, ... , b*_n_* relate to transformed age as previously described (Kemp et al. 2020):

$$F(chronological age)=b0+b1CpG1+...+bnCpGn+error$$

The DNAge^™^ estimate follows:

$$DNAge™=F(b0+b1CpG1+\ldots+bnCpGn)-1$$

This age prediction model was built initially starting with each of the sites present in Table S10, applying weight to each CpG site as described above, and in some instances that weight was zero; the weights applied to each CpG site are proprietary, therefore the exact subset of the sites used in the model (weights >0) is also proprietary.

**Statistics**

Body weight differences were assessed using a 2-tailed unpaired t-test. Tests for statistical significance in the promoter, exon(s), intron(s), rDNA, and mitochondrial DNA statistical analyses were done using a generalized linear model accounting for all groups and *p* values were controlled for false discovery using the Benjamini-Hochberg method. DNAge^™^ analysis differences were determined by comparing the aged PoWeR group to the aged sedentary group with a one-tailed t-test, since the data were normally distributed and homoscedastic, and the hypothesis was unidirectional.
